# Cingulate glutamate levels associate with pain in chronic pancreatitis patients

**DOI:** 10.1016/j.nicl.2019.101925

**Published:** 2019-07-02

**Authors:** Tine Maria Hansen, Janusiya Anajan Muthulingam, Asbjørn Mohr Drewes, Søren Schou Olesen, Jens Brøndum Frøkjær

**Affiliations:** a Mech-Sense, Department of Radiology, Aalborg University Hospital, Hobrovej 18-22, 9000 Aalborg, Denmark; b Department of Clinical Medicine, Aalborg University, Søndre Skovvej 11, 9000 Aalborg, Denmark; c Centre for Pancreatic Diseases, Department of Gastroenterology & Hepatology, Aalborg University Hospital, Mølleparkvej 4, 9000 Aalborg, Denmark

**Keywords:** Chronic pancreatitis, Magnetic resonance imaging, Spectroscopy, Glutamate, Pain, Metabolites

## Abstract

**Aims:**

Emerging evidence show that patients with chronic pancreatitis (CP) and abdominal pain have structural and functional alterations in the central nervous system. The aim was to investigate cerebral metabolic signatures in CP and the associations to various risk factors/clinical characteristics and patient outcomes.

**Methods:**

Magnetic resonance spectroscopy was used to measure brain metabolites in the anterior cingulate cortex (ACC), insula, prefrontal cortex and the parietal region in patients with CP and healthy controls. Subgroup analyses based on disease characteristics (alcoholic etiology of CP, diabetes and opioid treatment) were performed. Finally, relations to abdominal pain symptoms and quality of life scores were explored.

**Results:**

Thirty-one patients with CP (mean age 58.5 ± 9.2 years) and 23 healthy controls (54.6 ± 7.8 years) were included. Compared to healthy controls, patients had increased glutamate/creatine (glu/cre) levels in the ACC (1.24 ± 0.17 vs. 1.13 ± 0.21, *p* = .045) and reduced parietal *N*-acetylaspartate/creatine (NAA/cre) levels (1.44 ± 0.18 vs. 1.54 ± 0.12, *p* = .027). Patients with alcoholic etiology of CP had significant lower levels of parietal NAA/cre as compared to patients without alcoholic etiology and healthy controls (*p* < .006). Patients with a high level of ACC glu/cre reported more severe abdominal pain than their counterparts with a low level of ACC glu/cre (pain score 4.1 ± 2.7 vs.1.9 ± 2.3, *p* = .039).

**Conclusions:**

Cerebral spectroscopy revealed novel and complementary information on central pain mechanisms and alcohol mediated toxic effects in patients with CP. Our data suggest that cingulate glutamate levels associate with the patients clinical pain symptoms, while parietal NAA levels more likely associate with an alcoholic etiology of CP.

## Highlights

• Patients with chronic pancreatitis have altered brain metabolites.
• Increased cingulate glutamate levels associate with clinical pain symptoms.
• Decreased parietal *N*-acetylaspartate levels likely relate to alcoholic etiology.

## 1 Introduction

Chronic pancreatitis (CP) is characterized by long-standing inflammation of the pancreas resulting in morphological and functional alterations. Chronic abdominal pain is the hallmark symptom of CP and, in addition to changes in the pancreas and peripheral nerves, the chronic pain syndrome has been associated with an abnormal central pain processing and structural brain abnormalities (Olesen et al., 2013; Lelic et al., 2014; Dimcevski et al., 2007; Frøkjær et al., 2012; Frøkjær et al., 2011; Muthulingam et al., 2018). No published studies have, to the best of our knowledge, investigated whether cerebral metabolic alterations are present in patients with CP as compared to healthy subjects.

Magnetic resonance spectroscopy can be used to reveal brain metabolites such as *N*-acetylaspartate (NAA) recognized as a marker of neuronal functionality and density, creatine (cre) involved in neuronal energy metabolism, glutamate (glu) that is a neurotransmitter, myo-inositol (mI) that is a glial marker, and choline containing compounds associated with membrane turnover (Marjańska et al., 2017). These metabolites present potential indicators of neuronal dysfunction/alterations.

Magnetic resonance spectroscopy has previously been used to show that patients with chronic pain have a different neurometabolic status compared to healthy controls (Kameda et al., 2018; Ito et al., 2017; Feraco et al., 2011; Lv et al., 2018). Especially, higher levels of glu/cre and glx/cre (glx: glutamate and glutamine) in the anterior cingulate cortex (ACC) were demonstrated in chronic pain patients (Kameda et al., 2018; Ito et al., 2017). A study with Crohn's disease patients with abdominal pain also revealed higher levels of ACC glu/cre, than patients without abdominal pain and healthy controls (Lv et al., 2018). This indicates that magnetic resonance spectroscopy is a valid method to obtain information of pain related metabolic changes in the brain.

In this present study, magnetic resonance spectroscopy was used to investigate brain metabolite levels in patients with CP as compared to healthy controls. Although previous studies have shown that brain changes may be associated to chronic abdominal pain (Olesen et al., 2013; Lelic et al., 2014; Dimcevski et al., 2007; Frøkjær et al., 2012; Frøkjær et al., 2011; Muthulingam et al., 2018), it is likely that brain alterations could be caused by other CP related diseases characteristics, i.e. previous use of alcohol, diabetes, drug consumption, rather than chronic pain itself. Such factors need to be taken into consideration as alcohol, diabetes and opioids have previously been shown to be related to brain alterations (Selvarajah et al., 2011; Younger et al., 2011; Zahr and Pfefferbaum, 2017). We hypothesized that patient with CP have increased glu/cre in pain related areas and that this metabolite may be associated to patient outcomes (abdominal pain and quality of life). As an additional explanatory hypothesis, we explored the impact of other CP disease characteristics on brain metabolites in pain related areas and in white matter rich parietal region which might be vulnerable to changes due to alcohol exposure and diabetes (Zahr and Pfefferbaum, 2017; Hansen et al., 2019).

Thus, the aims were 1) to assess brain metabolites in patients with CP as compared to healthy controls in areas involved in pain processing (ACC, insula and prefrontal cortex) and in the parietal region, 2) to compare brain metabolite levels in subgroups with different disease characteristics (alcoholic etiology, diabetes, opioids treatment), and finally 3) to explore associations between altered brain metabolites in pain related areas and patient outcome (abdominal pain and quality of life).

## 2 Material and methods

### 2.1 Study population

Thirty-five patients with both painful and non-painful CP and 23 healthy controls were included in this cross-sectional study. The study was conducted at Centre for Pancreatic Diseases, Department of Gastroenterology and Hepatology and Department of Radiology, Aalborg University Hospital, Denmark. All subjects were screened by a medical doctor before enrollment. Patients were included if they had a CP diagnosis based on the Mayo Clinic diagnostic criteria (Layer and DiMagno, 1999) and exclusion criteria were: inability to undergo MRI, major illness such as cancer, pain syndromes other than CP and present alcohol or drug abuse. Furthermore, none of the patients had psychiatric illness, or used psychiatric medication. Age and gender matched healthy controls were included from our database. Healthy controls were without chronic pain conditions and any gastrointestinal symptoms. All subjects were informed about the study orally and in writing, and they provided informed written consent. Protocols were approved by the Ethics Committee of Northern Jutland (reference number N-20090008, N-20130040, N-20170023) and the study was conducted in accordance to the Declaration of Helsinki.

### 2.2 Disease characteristics

The electronic medical records were reviewed to obtain information on pharmacological treatment, diabetes status, and demographic characteristics including gender, age, etiology and duration of CP.

### 2.3 Magnetic resonance spectroscopy

**Fig. 1:**
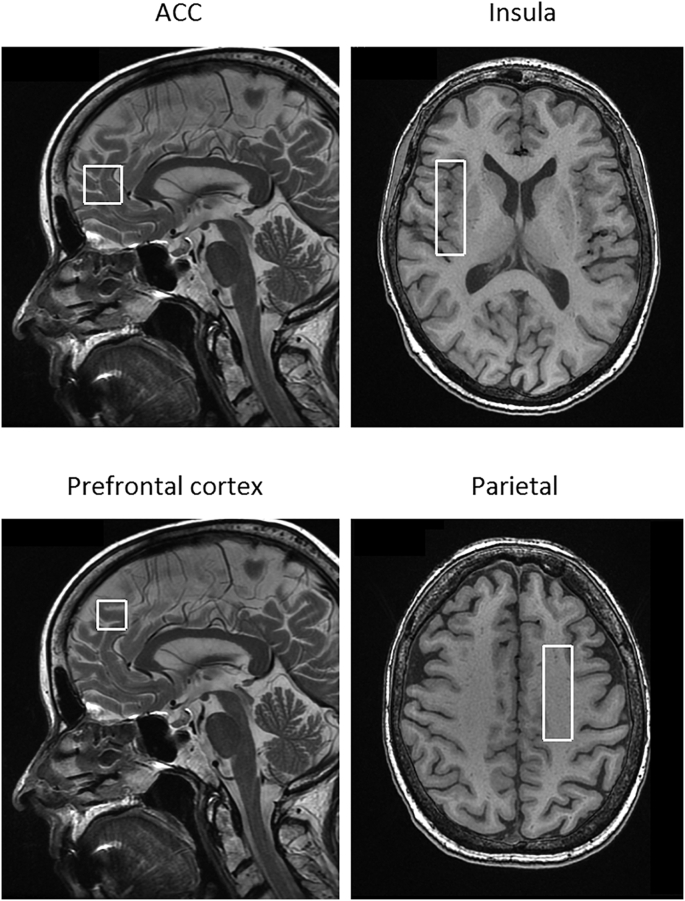
Positions of voxels of interests: Anterior cingulate cortex (20 × 20 × 20 mm), insula (15 × 20 × 50 mm), prefrontal cortex (15 × 15 × 20 mm) and the parietal region (15 × 15 × 50 mm).

Magnetic resonance spectroscopy measurements were obtained using a 3 T GE scanner (GE Signa HDxt, General Electric, Milwaukee, WI, USA). A standard eight-channel head coil was used and the head was fixed using foam pads. Single voxel PRESS (Point RESolved Spectroscopy) spectroscopy were acquired (TR/TE = 2000/30 ms) and total number of scans was 128 (5 min in total) in four different voxels of interests (VOIs), see example in Fig. 1. The VOIs were positioned in: 1) ACC (20 × 20 × 20 mm) on high resolution sagittal T2-weighted fast spin echo images in the midline of the pregenual ACC with the inferior border along the anterior-posterior commissure line, 2) right insula (15 × 20 × 50 mm) on high resolution axial T1-weigthed images and 3) the prefrontal cortex (15 × 15 × 20 mm) on the high resolution sagittal T2-weighted images, and 4) the parietal region (15 × 15 × 50 mm) contralateral to the side of the dominant hand, on the high resolution axial T1-weighted images and placed to cover as much white matter as possible.

A radiologist reviewed all scans for major structural changes within the VOIs. Potential scanner drift was monitored with a PRESS spectroscopy sequence of a phantom on each day.

LCModel (Version 6.3) was utilized for post-processing (Provencher, 1993). Water scaling and eddy-current correction were performed and metabolites were fitted in the chemical shift range 0.1–4.0 ppm. NAA/cre, glu/cre, mI/cre and GPC/cre were analyzed. Metabolites with Cramér-Rao bounds <30% were included. Quality measurements (signal-to-noise ratio (SNR) and full width at half maximum (FWHM)) for all spectra were provided from the analyses in LCModel.

### 2.4 Patient outcome measurements

Patient outcome measurements (abdominal pain and quality of life) were assessed using Brief Pain Inventory (BPI) short form (Mendoza et al., 2006) and the European Organization for Research and Treatment of Cancer Quality of Life Questionnaire Core 30 (EORTC-QLQ-C30) (Aaronson et al., 1993). The BPI questionnaire evaluates pain at its “worst”, “least”, “average”, and “now”. All four severity items were used to calculate the mean BPI pain score. Additionally, the BPI questionnaire measured how much pain has interfered with daily activities. The BPI pain interference is calculated as the mean of the seven interference items (Fayers et al., 2001a). EORTC-QLQ-C3O incorporates three main domains: Global health status, functional (physical, emotional, role, cognitive, social functioning) scales, and symptom scales (only the pain item was used). All three domains were analyzed according to the reference manual and presented as mean ± standard deviation. All three domains measure range in score from 0 to 100. A high score of global health status represents a high quality of life, a high score for a functional scale represents a healthy functioning, and a high score for a symptom scale represents a high level of symptomatology (Fayers et al., 2001b).

### 2.5 Statistical analyses

Differences of clinical characteristics and brain metabolite levels between patients and healthy controls were assessed using appropriate independent-sample *t*-tests or chi-squared tests. Only brain metabolites that were significant different between patients and healthy controls were investigated for further analyses regarding disease characteristics and patient outcomes.

Patient subgroups were stratified based on their 1) etiology of CP (alcoholic etiology, no/yes), 2) diabetes status (no/yes), and 3) current opioid treatment (no/yes). One-way ANCOVAs were conducted to analyze differences between patient subgroups and healthy controls controlling for age as covariate.

Patient reported outcomes (BPI pain and quality of life scores) were compared between patients with high and low glu/cre levels (stratified on the median metabolite level) using Mann-Whitney's *U* test. This was only explored in pain related areas that revealed changed metabolites.

Data are presented as mean ± standard deviation unless otherwise indicated and *p* < .05 was considered significant. Statistical analyses were performed in IBM SPSS Statistics (IBM Corp., Released 2017. IBM SPSS Statistics for Windows, Version 25.0. Armonk, NY, USA: IBM Corp.).

## 3 Results

Of the 35 patients considered for analysis, two patients were excluded due to poor MRI scan quality and two patients were excluded due to active alcohol abuse (revealed as ethanol peaks identified at 1.2 ppm in the metabolite spectra). Hence, thirty-one patients (mean age 58.5 ± 9.2 years) and 23 healthy controls (mean age 54.6 ± 7.8 years) were included for the final analysis. Spectroscopy data in the parietal region were for technical/logistic reasons only obtained from twenty-five patients. Demographics, clinical characteristics and brain metabolite levels are presented in Table 1. There were no significant differences in sex, age and body mass index (BMI) between patients and controls (all *p* ≥ .11). No significant differences in SNR or FWHM were found between spectra from the two groups in any of the four VOIs (all *p* > .19).

**Table 1:** Overview of demographical data, clinical data and metabolite concentrations.

	Patients with chronic pancreatitis	Healthy controls	*p*-value
	*n* = 31	*n* = 23	
Sex (M/F)	24/7	14/9	0.188
Age (years)	58.5 ± 9.2	54.6 ± 7.8	0.111
BMI (kg/m^2^)	23.7 ± 3.8	25.1 ± 2.5	0.129
Duration of CP (years)	10.3 ± 7.9		
Alcoholic etiology of CP (no/yes)	13/18		
Diabetes (no/yes)	18/13		
Pain status (no/yes)	9/22		
Opioid treatment (no/yes)	20/11		

Magnetic resonance spectroscopy ratios (no unit)			
ACC			
NAA/cre	1.04 ± 0.13	1.07 ± 0.14	0.415
Glu/cre	1.24 ± 0.17	1.13 ± 0.21 (n=22)	0.045*
mI/cre	0.77 ± 0.16	0.83 ± 0.23	0.328
GPC/cre	0.28 ± 0.03 (*n* = 29)	0.29 ± 0.06	0.332
Insula			
NAA/cre	1.27 ± 0.16	1.30 ± 0.15	0.475
Glu/cre	1.17 ± 0.21	1.13 ± 0.20	0.415
mI/cre	0.67 ± 0.14	0.74 ± 0.11	0.056
GPC/cre	0.23 ± 0.07 (*n* = 30)	0.24 ± 0.06	0.423
Prefrontal			
NAA/cre	1.28 ± 0.09	1.29 ± 0.13	0.771
Glu/cre	1.31 ± 0.22	1.27 ± 0.20	0.430
mI/cre	0.67 ± 0.13	0.72 ± 0.09	0.147
GPC/cre	0.25 ± 0.04 (*n* = 29)	0.24 ± 0.03 (*n* = 22)	0.180
Parietal			
NAA/cre	1.44 ± 0.18 (*n* = 25)	1.54 ± 0.12	0.027*
Glu/cre	0.91 ± 0.16 (*n* = 24)	0.87 ± 0.16 (*n* = 22)	0.414
mI/cre	0.83 ± 0.16 (*n* = 25)	0.87 ± 0.23	0.444
GPC/cre	0.33 ± 0.05 (*n* = 24)	0.34 ± 0.08	0.719

Quality measurements, SNR			
ACC	13.7 ± 3.9	14.6 ± 4.9	0.455
Insula	29.0 ± 6.1	28.2 ± 8.9	0.698
Prefrontal	13.6 ± 3.3	13.9 ± 2.5	0.716
Parietal	23.0 ± 4.2 (*n* = 25)	21.0 ± 6.5	0.191

Quality measurements, FWHM			
ACC	0.056 ± 0.016	0.052 ± 0.015	0.360
Insula	0.061 ± 0.010	0.058 ± 0.013	0.305
Prefrontal	0.040 ± 0.009	0.041 ± 0.013	0.807
Parietal	0.044 ± 0.012 (*n* = 25)	0.045 ± 0.009	0.768

Notes: Data are expressed as mean ± standard deviations unless otherwise stated. * indicates significant findings, **p<.05**. The number (*n*) of complete data are thirty-one and twenty-three for patients and healthy controls, respectively, unless otherwise stated in brackets.

Abbreviations: CP: chronic pancreatitis; M: males; F: females; BMI: body mass index; ACC: anterior cingulate cortex; NAA: *N*-acetylaspartate; cre: creatine; glu: glutamate; mI: myo-inositol; GPC: glycerophosphocholine; SNR: signal-to-noise ratio; FWHM = full width at half maximum (ppm).

### 3.1 Brain metabolites in patients with CP and healthy controls

Metabolite levels in the four regions of interest are presented in Table 1. Patients with CP had increased glu/cre levels in the ACC (*p* = .045) and reduced NAA/cre levels in the parietal region (*p* = .027) compared to healthy controls. Thus, subsequent subgroup analyses (section 3.2) were only done in these two areas and associations to patient outcomes were only explored for ACC glu/cre (section 3.3).

### 3.2 Brain metabolites in subgroups

**Fig. 2:**
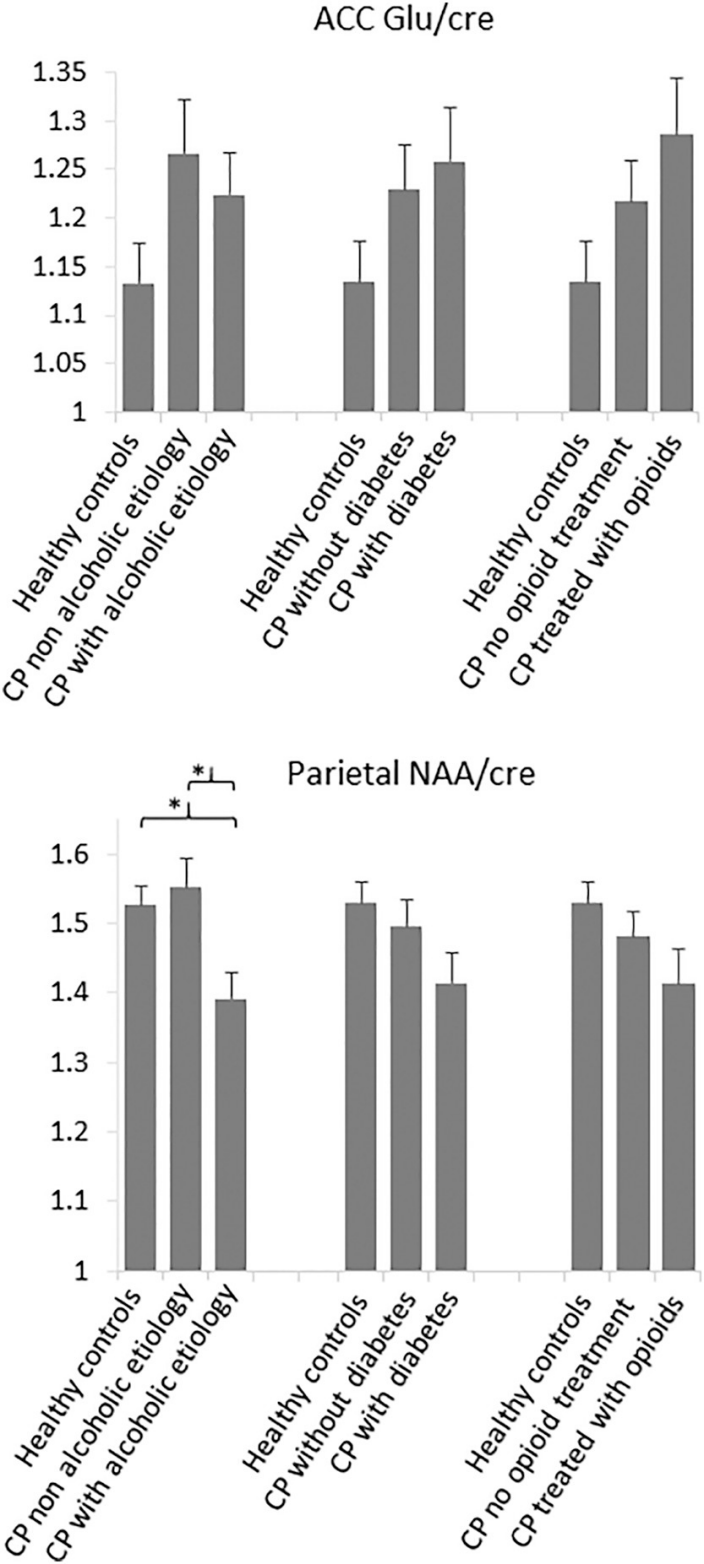
One-way ANCOVAs were conducted to analyze differences in ACC glu/cre (anterior cingulate cortex glutamate/creatine) and parietal NAA/cre (*N*-acetylaspartate/creatine) levels between patient subgroups and healthy controls. Patient subgroups were stratified based on their 1) etiology of CP (alcoholic etiology, no/yes), 2) diabetes status (no/yes), and 3) current opioid treatment (no/yes). * indicates significant findings.

Focusing on ACC glu/cre and parietal NAA/cre levels, a significant association between alcoholic etiology and NAA/cre levels in the parietal region (F = 5.78, *p* = .006) was found. As seen in Fig. 2, NAA/cre was lower in patients with alcoholic etiology of CP (*n* = 14) compared to patients with non-alcoholic etiology (*n* = 11) (*p* = .006) and healthy controls (*n* = 23) (*p* = .005). No differences were seen between patients without alcoholic etiology and healthy controls (*p* = .63). Furthermore, no differences were demonstrated in the subgroup analyses of parietal NAA/cre of diabetes (F = 2.39, *p* = .10) and opioid treatment (F = 1.95, *p* = .15). Finally, increased ACC glu/cre levels for patients were not associated with presence of alcoholic etiology of CP, diabetes or opioid treatment (all *p* > .10).

### 3.3 Brain metabolites in ACC and associations to patient reported outcomes

Patient reported BPI pain and quality of life scores for high and low metabolites levels of ACC glu/cre are presented in Table 2. Patients with highest glu/cre levels reported increased average pain scores (within the last 24 h) compared to their counterparts with lowest levels of glu/cre (*p* = .039) and felt worse pain (within the last 24 h) as compared to patients with lowest glu/cre (*p* = .046). Patients with high glu/cre had a trend towards decreased quality of life within the emotional functioning domain (*p* = .076). No significant differences were seen for other pain scores or quality of life domains (all *p* > .8).

**Table 2:** Patient reported outcome scores and high/low ACC glu/cre levels. The patient group were divided in high and low ACC glu/cre levels stratified on median level.

	ACC Glu/cre		p-value
	High	Low	
Pain scores (visual analogue scale 0–10)	(*n* = 14)	(*n* = 14)	
BPI pain	3.8 ± 2.7	1.9 ± 2.1	0.088
Pain right now	3.3 ± 3.0	1.9 ± 2.2	0.214
BPI interference	2.8 ± 2.0	1.8 ± 2.0	0.165
Pain worst 24 h	4.9 ± 2.9	2.6 ± 3.0	0.046*
Pain least 24 h	2.7 ± 2.8	1.1 ± 1.4	0.083
Pain average	4.1 ± 2.7	1.9 ± 2.3	0.039*

Quality of life (0−100)	(*n* = 13)	(*n* = 14)	
Global health	50.6 ± 24.6	63.7 ± 26.9	0.303
Physical functioning	69.2 ± 21.9	81.4 ± 21.3	0.160
Role functioning	64.1 ± 30.3	73.8 ± 31.8	0.327
Emotional functioning	58.3 ± 31.2	76.8 ± 32.9	0.076
Cognitive functioning	64.1 ± 33.2	75.0 ± 31.2	0.376
Social functioning	61.5 ± 35.6	81.0 ± 27.6	0.112
Pain	76.9 ± 54.4	48.2 ± 53.2	0.184

Notes: Data are expressed as mean ± standard deviations. * indicates significant findings, p<.05. BPI (Brief Pain Inventory) and quality of life questionnaires were missing due to non-compliance for three and four patients, respectively.

Abbreviations: ACC glu/cre; anterior cingulate cortex glutamate/creatine; *n*: number of patients included in the analysis, h: hours.

## 4 Discussion

In this study, magnetic resonance spectroscopy measurements were obtained from four brain areas in patients with chronic pancreatitis and healthy controls. Patients had increased levels of the excitatory neurotransmitter glutamate in the anterior cingulate cortex, which was associated with the clinical pain symptoms. Furthermore, as a marker of neuronal functionality patients had decreased white matter parietal *N*-acetylaspartate, which associated with an alcoholic etiology of CP.

### 4.1 Glutamate and associations to clinical outcomes

Increased glutamate levels in the ACC in patients with CP is a very relevant finding, since glutamate is an excitatory neurotransmitter which has previously been shown to be elevated in the ACC of patients with chronic pain (Ito et al., 2017). A previous study in patients with Crohn's disease revealed higher levels of ACC glu/cre in patients with abdominal pain that correlated with clinical pain scores (Lv et al., 2018). Along these lines, we demonstrated that CP patients with high ACC glutamate levels had increased pain symptoms and a trend towards decreased quality of life for especially the emotional functioning domain of the quality of life questionnaire. High ACC glutamate levels might indicate neuronal hyperexcitability, which is considered to be important in chronification of neuropathic pain (Boadas-Vaello et al., 2017). A previous pilot study showed that in CP patients responding to treatment with transcranial magnetic stimulation, the decrease in pain was correlated to changes in glutamate and NAA levels in the brain (Fregni et al., 2011). We found a higher glutamate level in patients with high pain as compared with those having low pain intensity, whereas such a correlation was not seen in the study by Fregni et al. (2011). Several methodological differences between the studies can, however, explain these differences, as brain metabolites were measured in SII and the transcranial magnetic stimulation induced increase in glutamate and NAA may be due to homeostatic mechanisms (Fregni et al., 2011).

One-third of the patients in our study were using opioids, which potentially could influence the measured metabolite levels. Especially, decreased levels of ACC glu/cre have been shown during acute morphine and oxycodone treatment (Hansen et al., 2016; Hansen et al., 2014). Thus, the glutamate level could potentially be underestimated for patients treated with opioids.

Previous electroencephalography studies suggested that patients with CP and abdominal pain symptoms have abnormal cerebral pain processing (Olesen et al., 2011) including functional reorganization in the cingulate and operculo-insular network (Lelic et al., 2014) and abnormal central excitability (Olesen et al., 2013). This present study demonstrates that also metabolic brain alterations in the ACC, potentially reflecting the functional changes due to long-lasting neuropathic-like pain mechanisms, may be associated to pain symptoms in patients with CP.

### 4.2 NAA and associations to alcoholic etiology of chronic pancreatitis

Previous studies in acute pain models and in chronic pain patients have revealed decreased NAA/cre levels in the ACC (Kameda et al., 2018; Hansen et al., 2014). In our study, NAA/cre levels were not altered in pain related areas (ACC, insula, prefrontal), but NAA/cre was decreased in the parietal region. This decrease in parietal NAA was associated to alcoholic etiology of CP. Since patients with alcoholic etiology had decreased NAA/cre as compared to patients without alcoholic etiology and healthy controls (and no difference was present between patients without alcoholic etiology and healthy controls) this could indicate that previously excessive consumption of alcohol might be a significant contributing factor for the decreased parietal NAA levels. NAA is known as a marker of neuronal functionality and density (Duarte et al., 2012) and magnetic resonance spectroscopy studies have shown lower levels of NAA in recently sober alcoholics relative to healthy controls in several brain regions (Zahr and Pfefferbaum, 2017). Regional cerebral alterations have mainly been reported in the frontal, temporal and parietal lobes in excessive alcohol consumers (Silveri et al., 2016). Magnetic resonance imaging studies have shown a specific vulnerability of white matter to chronic alcohol exposure (Zahr and Pfefferbaum, 2017), which imply the white matter rich parietal region to be more sensitive to alcohol related metabolic changes than gray matter rich brain areas. Alcohol-related CNS pathologies have shown white matter dysmyelination, demyelination and degeneration (de la Monte and Kril, 2014). Thus, the level of NAA in white matter may be related to the general condition of the myelinated axons.

Our data could support a theory that general structural brain changes and decrease of NAA could be more related to alcohol exposure (and other related co-factors), while functional brain alternations and regional glutamate changes are more related to pain mechanisms and the patients' clinical functioning.

### 4.3 Methodological considerations

Magnetic resonance spectroscopy measurements were obtained from predefined VOIs based on the literature, but other areas might also be relevant to investigate. As this was an explorative study, the significance level was not corrected for the number of VOIs or metabolites tested. Due to the relatively small number of subjects in the subgroup analyses, caution must be applied when interpreting the subgroup comparisons. Future studies should include more subjects or a study design to investigate specific subgroups including a more advanced multivariate statistical analysis. Finally, in this study, a few patients had incomplete data sets due to different reasons (non-compliance, data quality, etc.), and as several of the associations between the glutamate level and patient outcome pointed in a meaningful direction without being significant, it may indicate that more subjects will be needed in future studies to provide enough statistical power.

## 5 Conclusions

Brain metabolites were altered in patients with chronic pancreatitis. High level of glutamate in the anterior cingulate cortex was linked to increased perceived pain, which may indicate a hyperexcitatbility state of the anterior cingulate cortex in painful chronic pancreatitis. On the other hand, decreased levels of parietal NAA are likely more related to the general impact of alcohol on the brain and not chronic pancreatitis per se. A combination of structural, functional and metabolic brain assessments should be considered to further explore the multi-faceted role of the brain in patients with painful chronic pancreatitis.

## Funding

This study was supported by the Obel Family Foundation. The foundation had no further role in study design, in the collection, analysis and interpretation of data, in the writing the manuscript or in the decision to submit the paper for publication.

## Author contributions

TMH, JM, JBF acquired data; TMH, JM, SSO, AMD, JBF were involved in the study concept and design; TMH, JM, SSO, JBF analyzed the data; All co-authors were involved in interpretation of the data and critical revision of the manuscript.

## Declaration of Competing Interest

The authors declare that there is no duality of interest associated with this manuscript.
